# Paraspinal Muscle Fatty Infiltration as a Predictor of Intervertebral Disc Degeneration and Pain Severity in Chronic Low Back Pain

**DOI:** 10.1155/prm/1563996

**Published:** 2026-05-29

**Authors:** Weiyi Wang, Guanyi Wang, Jintao Dong, Simin Chi, Fang He, Xin Lei, Changchang Chen, Zhuojing Luo, Xiwen Liu, Bo Gao

**Affiliations:** ^1^ Department of Nursing, Air Force Medical University, Xi’an, 710032, China, fmmu.edu.cn; ^2^ Department of Orthopedics, Xijing Hospital, Air Force Medical University, Xi’an, 710032, China, fmmu.edu.cn; ^3^ Department of Anesthesiology, The 991st Hospital of Joint Logistic Support Force of People’s Liberation Army, Xiangyang, 441000, China; ^4^ General Surgery Department, The Second Medical Center & National Clinical Research Center for GeriAtric Diseases, Chinese PLA General Hospital, Beijing, 100853, China, 301hospital.com.cn; ^5^ The 941st Hospital of Joint Logistic Support Force of Chinese People’s Liberation Army, Xining, 810000, China; ^6^ School of Nursing and Rehabilitation, Xi’an FanYi University, Xi’an, 710105, China, xafy.edu.cn

**Keywords:** fatty infiltration, intervertebral disc degeneration, low back pain, paraspinal muscle, risk factor

## Abstract

**Background:**

Chronic low back pain (CLBP) is commonly associated with paraspinal muscle fatty infiltration and intervertebral disc degeneration (IDD); however, their independent contributions to pain and disability, as well as the influence of Body Mass Index (BMI) on these associations, remain unclear.

**Objective:**

This study aimed to investigate the associations of paraspinal muscle fatty infiltration rate (FIR) and cross‐sectional area (CSA)—specifically of the multifidus (MF), erector spinae (ES), and psoas major (PM)—with IDD severity, clinical metrics (pain (Visual Analog Scale [VAS]) and disability (Oswestry Disability Index [ODI])), and demographic factors (age, sex, and BMI) in patients with CLBP.

**Methods:**

A cross‐sectional study included 118 CLBP patients. FIR and CSA of paraspinal muscles at L2–S1 were quantitatively measured on MRI using ImageJ. IDD was graded using the Pfirrmann system. Correlations and group comparisons were statistically analyzed.

**Results:**

FIR showed significant sex‐based differences but no left–right asymmetry and was not associated with BMI. MF FIR was strongly positively correlated with Pfirrmann grade (*r* > 0.7, *p* < 0.05). FIR of MF and ES was positively correlated with age and disease duration, as well as VAS and ODI scores (*r* > 0.6, *p* < 0.05), whereas CSA showed only weak correlations. The PM exhibited minimal associations with clinical or degenerative parameters.

**Conclusion:**

Paraspinal muscle fatty infiltration, especially in the MF and ES, is strongly associated with IDD severity, pain, and disability independently of BMI. FIR could serve as a potential imaging biomarker for assessing CLBP severity and progression.

## 1. Introduction

Chronic low back pain (CLBP), accounting for approximately 23% of all low back pain cases, remains etiologically unclear [1] and may be closely associated with impaired spinal stability [2, 3]. In the functional spinal unit (FSU), intervertebral discs bear axial compressive loads, while paraspinal muscle activity reduces disc static loading by approximately 40% and mitigates abnormal torsional and shear stresses. The interaction between intervertebral discs and paraspinal muscles contributes to the maintenance of spinal stability. Intervertebral disc degeneration (IDD) and paraspinal muscle fatty infiltration are commonly associated in CLBP [4, 5], but the relationship between IDD, fatty infiltration, and pain is unclear.

IDD arises from multiple etiological factors, including abnormal loading, aging, and injury [6–8]. The decline in disc cushioning and spinal stability caused by IDD is an irreversible process. A growing body of evidence indicates a close association between IDD and paraspinal muscle [9]. Paraspinal muscle mainly consists of multifidus (MF), erector spinae (ES), and psoas major (PM) muscles, and its degeneration is mainly in the form of muscle atrophy and fatty infiltration. As in other organs, fatty infiltration in paraspinal muscles induces structural alterations and compromises their normal function [10, 11]. Previous research has established a link between severe IDD and fatty infiltration in the MF and ES muscles, often accompanied by endplate changes [12]. However, the precise relationship between IDD, paraspinal muscle fatty infiltration, and pain remains unclear.

Fatty infiltration in skeletal muscle may lead to chronic low‐grade inflammation, which disrupts redox homeostasis and promotes the differentiation of interstitial mesenchymal stem cells into adipocytes rather than myocytes. These processes further enhance macrophage activation, stimulate proinflammatory cytokine release, and exacerbate adipocyte accumulation, collectively contributing to the deterioration of skeletal muscle structure and function [13, 14].

Fatty infiltration is measured using visual assessment, semiquantitative assessments such as Goutallier grading, and diffusion tensor imaging [15, 16] currently. In contrast, ImageJ‐based quantification of FIR—derived from muscle cross‐sectional area (CSA) and fat area measurements in magnetic resonance images (MRIs)—offers a highly operable, noninvasive, and quantitative alternative with advantages including high resolution, low radiation exposure, and ease of use.

This study used Image J software to measure the FIR of MF, ES, and PM muscles at L2–S1 segments. The study investigated the relationship between FIR and IDD, while analyzing its correlations with age, sex, Body Mass Index (BMI), Visual Analog Scale (VAS) scores, and Oswestry Disability Index (ODI). Furthermore, the characteristics of FIR and its relationship with low back pain severity, functional impact, and IDD were explored. The central hypothesis of this study is that fatty infiltration in paraspinal muscles is positively associated with three key factors: pain intensity (VAS scores), functional impairment (ODI), and the extent of IDD.

## 2. Materials and Methods

### 2.1. Participants

A total of 118 CLBP patients who received treatment in the First Affiliated Hospital of Air Force Medical University from October 2023 to July 2024 were enrolled. Their medical records and complete imaging data were retrospectively reviewed and collected.

Inclusion criteria are as follows: (1) Duration of low back pain more than 3 months. (2) Age 18–80 years.

Exclusion criteria are as follows: (1) Patients with other spinal conditions, including neoplasms, tuberculosis, spinal deformities, spinal stenosis, lumbar disc herniation (as the study focuses on degeneration rather than herniation), spondylolisthesis, or lumbar fractures. (2) Individuals with any history of spinal surgery or trauma. (3) Those with systemic diseases affecting muscle metabolism or inflammatory status, such as diabetes, thyroid dysfunction, Cushing’s syndrome, rheumatic diseases (e.g., rheumatoid arthritis and ankylosing spondylitis), or active tuberculosis. (4) Pregnant or postpartum women, or individuals with claustrophobia. (5) Individuals who have undergone systematic lower back muscle training within the past 3 months. (6) Those who received interventional treatments for lumbar spine conditions (e.g., epidural injections and radiofrequency ablation) within the last 6 months. (7) Individuals who declined to participate in the study.

This study is a retrospective cross‐sectional analysis, with the sample size determined by the number of consecutive patients with CLBP who attended our department between October 2023 and July 2024 and met all inclusion and exclusion criteria.

### 2.2. Information Collection

Patient demographic data, including sex, age, height, and weight, were collected. The degree of IDD was graded using the Pfirrmann grading system. Pain severity was assessed using the VAS, and the extent of functional disability was evaluated using the ODI.

### 2.3. Image Analysis

#### 2.3.1. Measurements of the CSA and FIR

A detailed analysis of T2‐weighted MRI was performed at the upper endplate level of lumbar segments L2–S1 in the axial plane. Using ImageJ (Version 1.53a, National Institutes of Health, USA), the MF, ES, and PM muscles were quantitatively assessed at each segment. Muscle and fat boundaries were manually delineated for the L2/3, L3/4, L4/5, and L5/S1 segments (Figure 1). The CSA was defined as the total anatomical area of the PSM, including both muscle and intramuscular fat tissue, whereas the fat replacement area referred specifically to the adipose tissue within the defined CSA. All measurements were performed in triplicate and averaged. The FIR was calculated as (fat replacement area/CSA) × 100%.

FIGURE 1MF as an example of CSA and fat replacement area measurement using ImageJ software. (a) ImageJ software to measure CSA of MF. (b) ImageJ software to measure muscle replacement area in MF.

**Figure:**
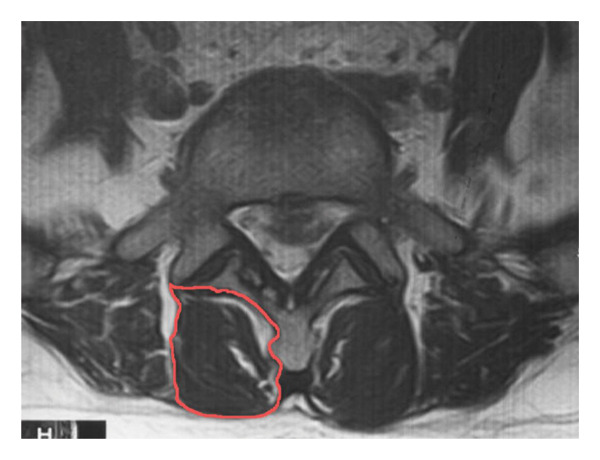
(a)

**Figure:**
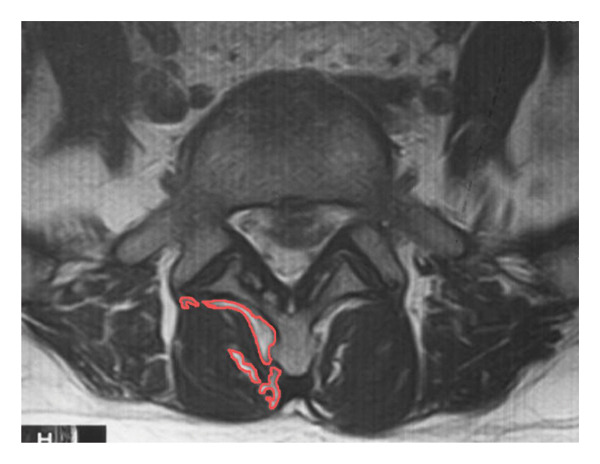
(b)

This study did not perform measurement analysis at the L1/2 segment, primarily due to the following reason: based on the typical scanning range and anatomical structures of the MRI sequence employed in this study, the paraspinal muscles at the L1/2 level, particularly the MF, often exhibit incomplete muscle boundaries and blurred contours on transverse images. This makes it difficult to ensure the accuracy and consistency of manual contouring measurements.

#### 2.3.2. Assessment of IDD

**FIGURE 2:**
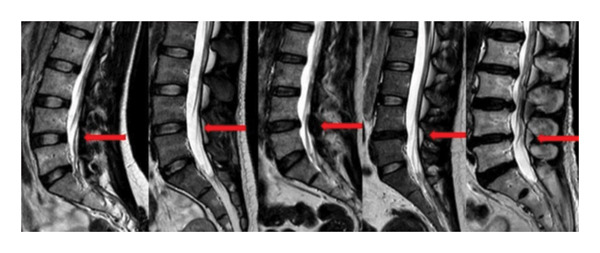
The images from left to right correspond to Pfirrmann Grades I through V.

IDD severity was graded on midsagittal T2‐weighted MRI according to the original Pfirrmann grading system by two fellowship‐trained spine surgeons with more than 5 years of clinical experience (Figure 2). Grading was performed independently, and any discrepancies were resolved by consensus discussion.

### 2.4. Statistical Analysis

Statistical analysis was performed using SPSS 26.0. Normally distributed continuous variables (e.g., age) were expressed as mean ± standard deviation and compared using the independent samples *t*‐test. Nonnormally distributed data were summarized as median (Q1 and Q3) and analyzed with the Mann–Whitney *U* test. Categorical variables were described as frequency (percentage) and compared using the chi‐square test. For correlation analysis, Pearson’s test was applied for normally distributed continuous variables, while Spearman’s rank correlation was used for nonnormal distributions. A *p* value < 0.05 was considered statistically significant for all analyses.

This study is a retrospective cross‐sectional analysis, and the sample size was determined by the number of consecutive patients with CLBP who attended our department during the study period and met all inclusion and exclusion criteria. No prospective power analysis was performed.

## 3. Results

### 3.1. Patient Characteristics

A total of 118 patients with CLBP were included in this study(72 males and 46 females). The details are shown in Table 1.

**TABLE 1:** Patient demographics and characteristics.

Variables (*n* = 118)	Category	Frequency/*M* ± SD	Percentage
Sex	Male	72	61.0
	Female	46	39.0

Age (year)		52.42 ± 13.78	

BMI (kg/m^2^)		24.74 ± 3.15	

Disease course (months)		59.24 ± 32.12	

Occupation	Farmer	50	42.4
	Civil servant/office worker	46	39
	Retired	7	5.9
	Self‐employed	12	10.2
	Unemployed	2	1.7
	Worker	1	0.8

Residence	Urban	67	56.8
	Rural	51	43.2

Education level	Primary school or below	30	25.4
	Junior high/secondary vocational	30	25.4
	Senior high/junior college	33	28.0
	Bachelor’s degree or above	25	21.2

Marital status	Married	101	85.6
	Single	8	6.8
	Divorced	5	4.2
	Widowed	4	3.4

Smoking history	Yes	39	33.1
	No	79	66.9

Alcohol consumption history	Yes	28	23.7
	No	90	76.3

Abbreviation: BMI, body mass index.

### 3.2. Pfirrmann Grading Distribution of IDD

**FIGURE 3:**
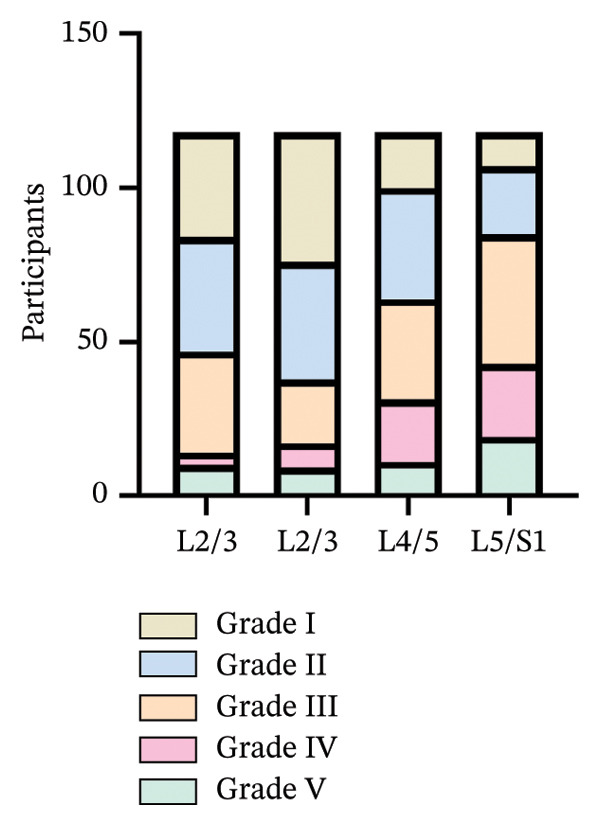
Distribution of different Pfirrmann grades in each segment (*n* = 118).

Degenerative changes of Grades II and III were relatively prevalent, followed by Grade I, while Grades IV and V degeneration were comparatively less frequent (Figure 3).

### 3.3. Difference of FIR in Pfirrmann Grading of IDD in Each Segment

#### 3.3.1. MF

One‐way ANOVA revealed significant differences in the FIR of the MF muscle across most paired comparisons of Pfirrmann grades at the L2–S1 segments, *p* < 0.05. The exceptions were no significant differences between Grades I and II at the L2/3, L4/5, and L5/S1 segments and between Grades II and IV at the L2/3 and L5/S1 segments (Figure 4(a)).

FIGURE 4Differences in FIR among different Pfirrmann gradings in L2–S1 segments of paraspinal muscles (*n* = 118). (a) Difference of FIR in different Pfirrmann grading in MF L2–S1 segments. (b) Differences in FIR in ES L2–S1 segments with different Pfirrmann grading. (c) Differences in FIR in PM L2–S1 segments with different Pfirrmann grading. FIR: fatty infiltration rate.

**Figure:**
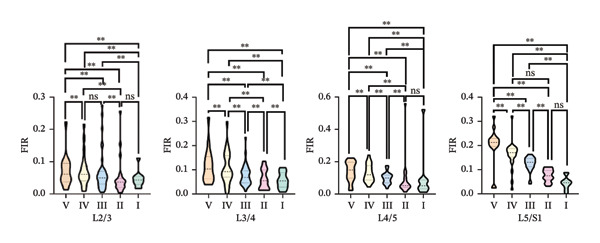
(a)

**Figure:**
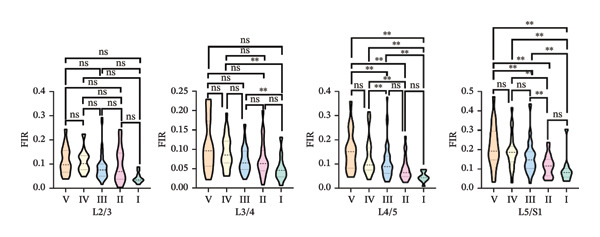
(b)

**Figure:**
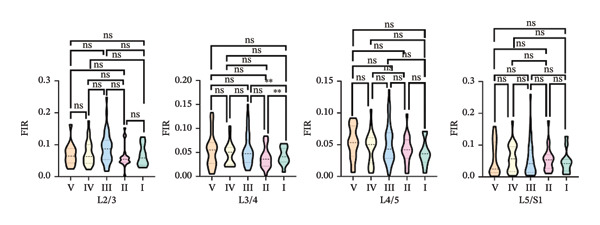
(c)

#### 3.3.2. ES

One‐way ANOVA indicated significant differences in FIR between nearly all paired Pfirrmann grades at the L2–S1 segments, *p* < 0.05. Nonsignificant differences were observed for all grade comparisons at the L2/3 segment; between Grades I vs. III, I vs. IV, I vs. II, II vs. III, and IV vs. V at the L3/4 segment; and between Grades I vs. II, III vs. IV, and IV vs. V at the L5/S1 segment (Figure 4(b)).

#### 3.3.3. PM

One‐way ANOVA revealed that no significant differences were observed in the PM across the L2–S1 segments at any Pfirrmann grade, *p* > 0.05, with the exception of significant differences between Grades I and II and between Grades I and III at the L3/4 segment, *p* < 0.05 (Figure 4(c)).

### 3.4. Symmetrical Distribution of Fatty Infiltration in Bilateral Paraspinal Muscles

The Mann–Whitney *U* test revealed no statistically significant differences in the FIR between the left and right sides for the MF, ES, and PM muscles across all measured segments, *p* > 0.05, with the exception of the PM at the L5/S1 segments (Figures 5(a), 5(b), 5(c)).

FIGURE 5Differences in FIR of paravertebral muscles at L2–S1 segments between sex, left and right sides of the spine (*n* = 118). (a) Comparison of the differences in FIR between the left and right sides of the MF at the L2–S1 segments. (b) Comparison of the difference in FIR between the left and right sides of ES in L2–S1 segments. (c) Comparison of differences in FIR between left and right sides of PM in L2–S1 segments. (d) Differences in MF FIR of L2–S1 segments between sex. (e) Differences in ES FIR in L2–S1 segments between sex. (f) Differences in PM FIR at the L2–S1 segments between sex. FIR: fatty infiltration rate.

**Figure:**
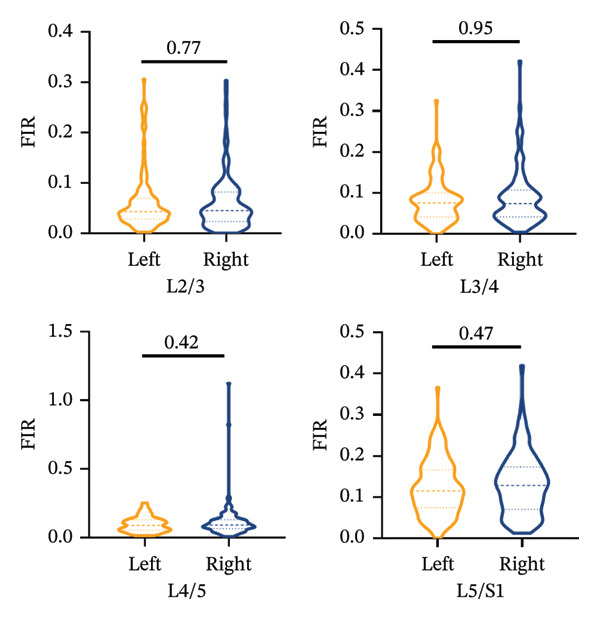
(a)

**Figure:**
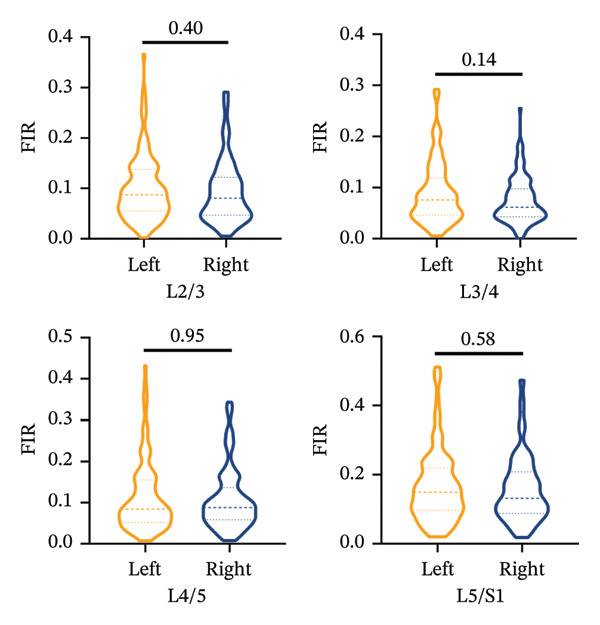
(b)

**Figure:**
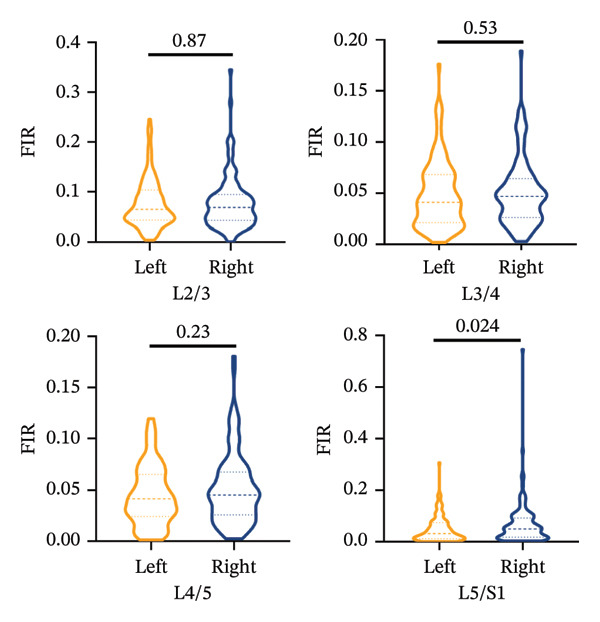
(c)

**Figure:**
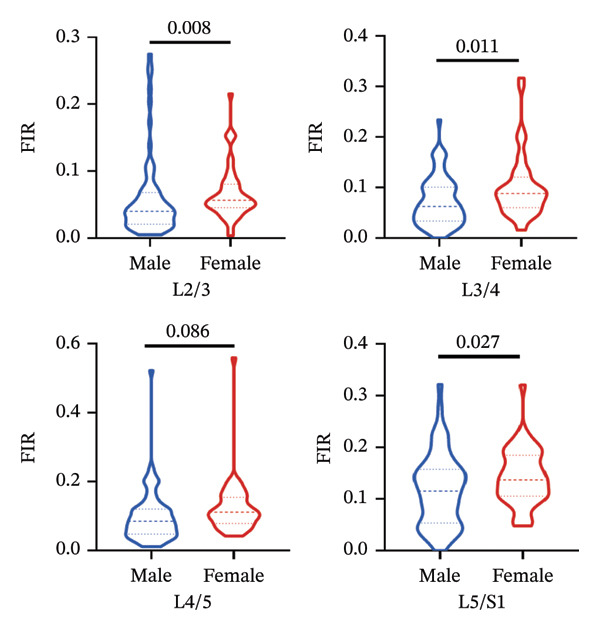
(d)

**Figure:**
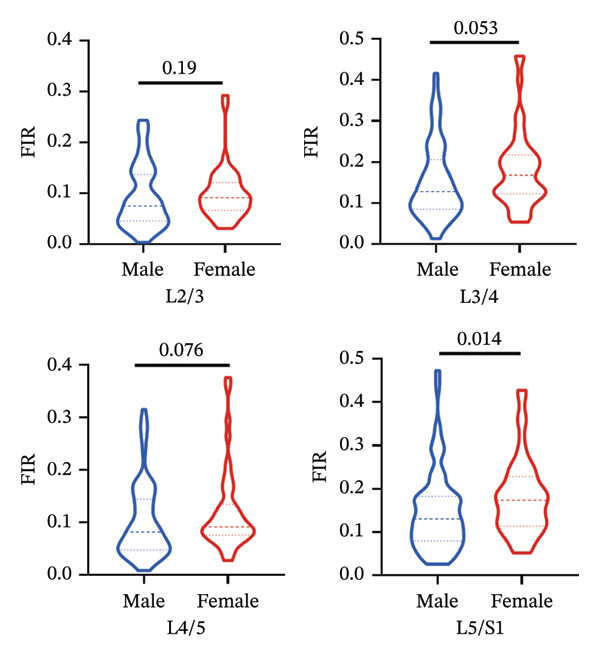
(e)

**Figure:**
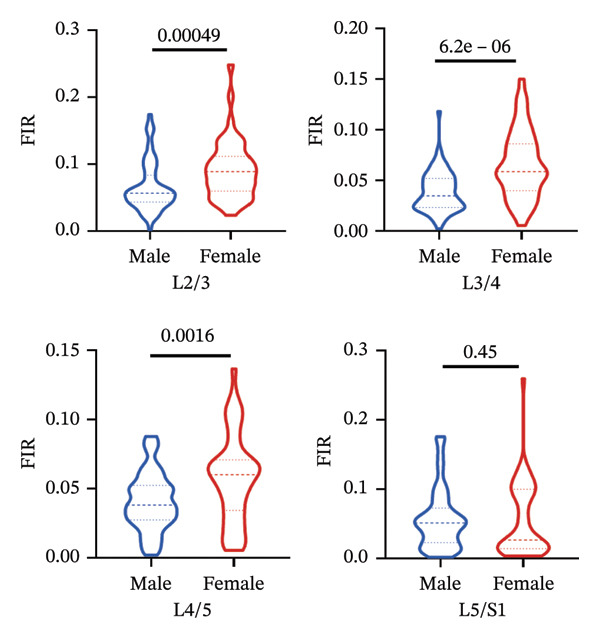
(f)

### 3.5. Sex‐Related Variations in Fatty Infiltration Across Spinal Segments

#### 3.5.1. MF

Mann–Whitney *U* tests revealed significant sex differences in the FIR of the MF at all examined segments (L2/3, L3/4, L4/5, and L5/S1), *p* < 0.05 (Figure 5(d)).

#### 3.5.2. ES

Significant sex‐based differences in FIR were observed in the ES across most lumbar segments (L3/4, L4/5, and L5/S1), *p* < 0.05, with the exception of the L2‐3 segment, which showed no significant difference (Figure 5(e)).

#### 3.5.3. PM

Sex differences in FIR were identified in the PM at the L2/3 and L3/4 segments, *p* < 0.05. However, no significant differences were found at the L4/5 and L5/S1 segments (Figure 5(f)).

### 3.6. Association Between IDD and Paraspinal Muscle FIR

**FIGURE 6:**
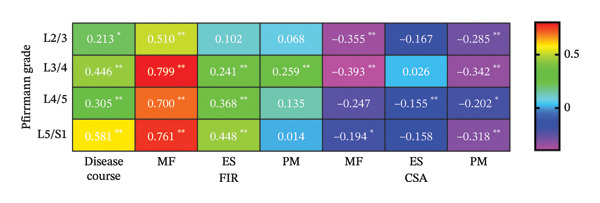
Correlation between FIR, CSA, disease duration, and Pfirrmann grading of MF, ES, and PM (*n* = 118). MF: multifidi; ES: erector spinae; PM: psoas major; FIR: fatty infiltration rate; CSA: cross‐sectional area.  ^∗^ *p* < 0.05,  ^∗∗^ *p* < 0.01.

Spearman correlation showed strong positive associations between FIR and Pfirrmann grade in the MF at all L2–S1 segments and in the ES at L3–S1; the PM exhibited a significant correlation only at L3/4, *p* < 0.05 (Figure 6).

### 3.7. Association Between IDD and Paraspinal Muscle CSA

Spearman correlation showed CSA to correlate weakly and negatively with Pfirrmann grade in both MF and PM at all L2–S1 segments, *p* < 0.05. No significant association was detected in ES at any segment, *p* > 0.05 (Figure 6).

### 3.8. Association Between IDD and Disease Duration

Spearman’s correlation analysis revealed significant moderate positive associations between the fatty infiltration rate (FIR) and Pfirrmann grade at all examined lumbar segments, *p* < 0.001 (Figure 6).

### 3.9. Association of Paraspinal Muscle FIR With Clinical and Demographic Variables

**FIGURE 7:**
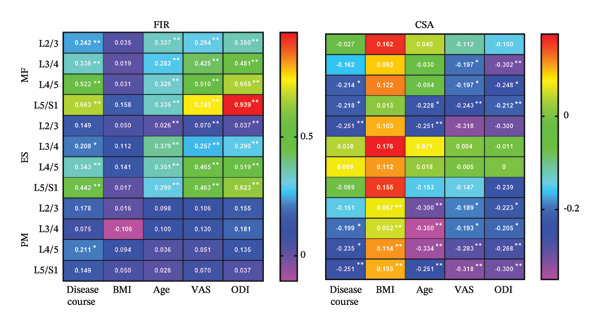
Correlation of FIR, CSA with disease duration, BMI, age, VAS, and ODI in MF, ES, and PM (*n* = 118). MF: multifidi; ES: erector spinae. PM: psoas major. FIR: fatty infiltration rate. CSA: cross‐sectional area. BMI: Body Mass Index. VAS: Visual Analog Scale. ODI: Oswestry Disability Index.  ^∗^ *p* < 0.05,  ^∗∗^ *p* < 0.01.

Spearman correlation showed that in the MF disease duration correlated positively with FIR at all L2–S1 segments, with the strength increasing caudally, *p* < 0.01. In the ES, a significant positive association was present at L3‐S1, *p* < 0.01, but not at L2/3. For the PM, only the L4/5 segment exhibited a weak correlation, *p* < 0.05, while all other levels were nonsignificant. FIR in MF and ES was unrelated to BMI, *p* > 0.05, but correlated strongly with VAS and ODI scores, *p* < 0.01 (Figure 7).

### 3.10. Association of Paraspinal Muscle CSA With Clinical and Demographic Variables

Spearman analysis revealed weak negative correlations for CSA with disease duration at L3–S1 in both MF and PM, *p* < 0.05. Similarly, CSA in MF (L4–S1) and PM (L2–S1) correlated weakly with age, *p* < 0.05. Low‐strength negative associations were also present between CSA and VAS/ODI scores in MF (L3–S1) and PM (L2–S1), *p* < 0.05 (Figure 7).

## 4. Discussion

CLBP imposes a substantial healthcare burden [17] and markedly compromises patients’ daily activities and work capacity [18]. Paraspinal muscles are core structures that maintain spinal stability and protect against IDD. Fatty infiltration, as one of the degenerative manifestations of these muscles, compromises their structural integrity, thereby impairing lumbar function, restricting physical mobility, inducing pain, and diminishing quality of life (3). Previous studies have explored correlations between paraspinal muscle fatty infiltration and factors such as IDD, age, and BMI [19]; however, these investigations were limited by small sample sizes, narrow focus on specific muscles, and spinal segments, and yielding incomplete and coarse data. Using ImageJ, we quantified the fat and CSAs of the MF, ES, and PM at each lumbar segment from L2–S1, conducting a detailed analysis of the correlations between FIR and variables including age, sex, anatomical level, degree of IDD, BMI, VAS scores, and ODI scores.

Among the four disc segments observed in this study, Grades II and III degeneration accounted for more than Grade V; FIR was not the same for different IDD degrees, which is consistent with the results of previous studies [20], but ES and PM were not analyzed in these studies. Some scholars compared the CSA of paraspinal muscles between patients with unilateral pain in CLBP and asymptomatic individuals, and the CSA area of the former decreased and was positively correlated with pain and duration [21]. In the study of Sudhir et al. [22], IDD often coexists with paraspinal muscle fiber irregularities (FIR) and even a vicious cycle may occur. Paraspinal muscle degeneration causes spinal imbalance, leading to improper load distribution on intervertebral discs and the occurrence of IDD. Our research results can establish a connection between FIR and both the prediction and grading of IDD.

The present study demonstrated symmetric FIR in MF, ES, and PM between left and right sides at L2–S1. Asymmetric degeneration of the paraspinal muscles has been previously reported in patients with unilateral back pain, adolescent and adult scoliosis, and lumbar disc herniation [23, 24]. In contrast, we provide novel evidence that paraspinal muscle FIR progresses symmetrically in CLBP patients, independent of the laterality of pain. The phenomenon of differences in the FIR of paravertebral muscles on both sides of the spine has only been reported in adolescent idiopathic scoliosis and degenerative scoliosis [25]. In a study of healthy individuals, differences in the CSA of paravertebral muscles, especially the MF, have been proposed as a possible indicator of CLBP or spinal lesions [26]. The degeneration of ES in healthy adults is asymmetric, regardless of handedness, with the CSA of the left MF being larger than that of the right side [27]. Some studies suggest that the asymmetry of paraspinal muscle degeneration may be related to lifestyle rather than caused by spinal diseases [28]. Contrary to the scarcity of data on side‐to‐side fatty infiltration in CLBP, we provide the first evidence that paraspinal FIR progresses almost symmetrically in CLBP, with no left–right discrepancy and no correspondence to the self‐reported pain side. FIR shows an upward trend with the variation of stress within the segment.

Consistent with prior reports of higher female FIR of MF and ES [29], we extended the analysis to PM and bilateral compartments, confirming the sex effect across all three paraspinal muscles. In addition, we observed that the left, right, and total CSA of PM exhibited no sex difference exclusively at L5/S1, whereas ES exhibited the same pattern only at L2/3. Notably, MF is more susceptible to pain‐mediated inflammatory and immune responses—likely due to its immediate proximity to the spinous processes and extensive fascial attachments—which can lead to atrophy and fatty infiltration [30].

The phenomenon of FIR increasing with age has been observed in many organs infiltrated by fat [4, 31–33]. Shahidi et al. [34] found that the FIR of MF and ES increased with age, but the change of muscle CSA was not significant, especially in females. They suggested that the potential mechanism might be related to postmenopausal hormonal deficiency, which contributes to the decline of muscle function [35]. This suggests that the degeneration of MF and ES is dominated by fatty infiltration and exhibits sex‐specific differences. Following the work of Cordani et al. [36] in mice and subsequent human studies on age‐related fat increases in the MF and ES [37–40], we extended the analysis to PM FIR and CSA in an expanded patient cohort. The present study extended the analysis to include PM FIR and CSA in relation to age and enrolled a larger patient cohort. Our analysis revealed weak positive correlations between age and FIR in the MF and ES. In contrast, while PM FIR showed no significant correlation with age, PM CSA exhibited a weak negative correlation with age. These findings support the hypothesis that the PM may compensate for degenerative changes in the MF and ES. Specifically, as fatty infiltration leads to degeneration of the MF and ES—compromising disc stability and contributing to lower back pain—the PM may play an increasingly important role in stabilizing the lumbar spine and maintaining lordosis [41].

In a cross‐sectional study [42], patients were categorized into acute, chronic local, and chronic widespread pain groups according to the duration and extent of back pain, and significant body composition differences were found in the chronic group compared with the pain‐free control group, a phenomenon that was particularly pronounced in patients with widespread pain who had a much longer duration of disease. Our study verified that there is an association between pain duration and fatty infiltration, but causality and specific mechanisms need to be further explored [14, 43, 44].

There have been studies that graded fatty infiltration in MF using semiquantitative methods and found that the area of fatty infiltration in MF was not affected by BMI [45], but in a study by Seyedhoseinpoor et al. [46], BMI may be a confounding factor for fatty infiltration. FIR of paraspinal muscles in the neck has also been shown to be independent of BMI and cervical disc degeneration [47]. In the present study, FIR of MF, ES, and PM was also not associated with BMI. Higher BMI has been shown to be causally related to the risk of disc degeneration and CLBP [48], but fat is not uniformly deposited in different disc segments but varies with disc stress, as it did in our results, so we are inclined to accept the finding that BMI is not related to fatty infiltration. Research suggests that localized fat distribution indices, such as the subcutaneous fat index (SFI) at the L1‐L2 level, may hold greater predictive value than BMI for lumbar spinal degeneration and pain [49]. The SFI reflects localized metabolic and mechanical environments, potentially exerting a more direct influence on pathological processes within paraspinal tissues. Future investigations into the relationship between adiposity and spinal health should, therefore, consider both systemic obesity indicators (e.g., BMI) and localized fat distribution metrics (e.g., SFI and visceral fat area) to achieve a more comprehensive understanding of adipose tissue’s role in the pathophysiology of CLBP.

The study explored the relationship between low back pain and FIR and CSA. The results showed that in MF and ES, FIR gradually increased with lumbar spine stress from top to bottom at L2–S1, and VAS and ODI showed the same trend with stress, and the correlation with FIR was enhanced with the increase of stress in the lumbar spine segments. We found that the FIR of PM, on the other hand, was not related to VAS and ODI, a result that is consistent with previous studies [50, 51]. CSA did not show a similar correlation with VAS and ODI. As an important endocrine organ in the human body, adipose tissue can secrete adipokines to regulate lipid metabolism, inflammatory response, and tissue sensitivity to insulin [52]. By establishing animal models of disc degeneration and spinal injury, it was possible to reveal that there is dysregulation of inflammatory mediators and inflammatory factors involved in adipose infiltration in MF [43]. There is a subjective element in the pain measurements currently used [53, 54], and the more commonly used pain assessments are the VAS, NRS, and VRS, as well as the McGill Pain Questionnaire and the Pain‐O‐Meter Pain Instrument, which more accurately differentiate between the effects of pain [55, 56], all of which have not only the advantage of good reliability [57] but also suffer from the problems of subjectivity, dependence on the place, and time‐consuming. Lentini et al. [58] have pointed out that in future, in athletic populations, cardiovascular disease will be a major problem. In the future, in the athlete population, changes in cardiovascular variables may reflect the degree of pain; a combination of molecular, neuroimaging, and sensory markers shows potential in the classification of TMJ disorder pain [59]. Our finding that paraspinal muscle CSA was not associated with pain in the lower back pain population corroborates with the study by Cankurtaran et al. [60]. FIR of MF and ES was highly correlated with patients’ VAS and ODI, a finding that suggests a possible link between subjective indicators used to measure low back pain and paraspinal muscle FIR directly.

It is noteworthy that in recent years, researchers have proposed a comprehensive scoring system—the “Mo‐fi‐disc” score [61]—combining MRI paraspinal muscle fatty infiltration, IDD, and clinical symptoms. This system, which integrates radiographic changes with clinical phenotypes, has been demonstrated to effectively distinguish patients with low back pain from asymptomatic individuals, with a threshold of 5.5 [62]. The findings of this study provide important quantitative evidence for the biological rationale of such a composite scoring system: we observed strong correlations between MF and ES FIR and Pfirrmann grading, alongside significant positive correlations with VAS and ODI scores. This suggests a close intrinsic relationship between fatty infiltration, disc degeneration, and clinical symptoms, supporting the necessity of integrating them into a multidimensional assessment metric. Future research may further explore incorporating the quantitative FIR measurement employed in this study into such composite scoring systems, aiming to achieve more precise assessment and risk stratification of CLBP severity.

Consistent with a recent study [63], our research found that the lower lumbar segments (L4–S1) of both CLBP patients have significant MF fatty infiltration; in addition, we further found that CLBP patients have mild ES fatty infiltration at the upper lumbar segment, and the infiltration degree is positively correlated with disease duration, which verifies the specificity of upper lumbar ES fatty infiltration in CLBP patients compared with asymptomatic subjects.

In summary, our findings demonstrate a symmetric distribution of fatty infiltration between the left and right sides of the paraspinal muscles. However, significant differences were observed based on sex and age but not with BMI. Furthermore, fatty infiltration was positively correlated with the severity of pain, functional disability (as measured by VAS and ODI) and the degree of IDD (Pfirrmann grade). These correlations were most pronounced in the MF muscle.

Novelty and contributions to the literature: The present study has several novelties and strengths that provide important additions to the current literature.

First, this study simultaneously and quantitatively evaluated the FIR of the MF, ES, and PM muscles at the L2–S1 segments, which provides a more comprehensive understanding of paraspinal muscle degeneration in patients with CLBP. Second, this study confirmed that fatty infiltration of the paraspinal muscles was strongly associated with disc degeneration, pain intensity, and functional disability independently of BMI, which helps clarify the unique role of muscle fatty degeneration in the pathophysiology of CLBP. Third, this study demonstrated that fatty infiltration was symmetric between the left and right sides in patients with CLBP, which provides a new insight into the distribution characteristics of paraspinal muscle degeneration. Fourth, this study verified that the FIR is a more reliable imaging biomarker for evaluating low back pain and disc degeneration than muscle CSA.

These findings improve the current understanding of the interaction between paraspinal muscle degeneration and IDD and provide valuable imaging indicators for the clinical assessment and risk stratification of CLBP.

### 4.1. Limitations

Due to time and material constraints, this study was not able to include more possible influencing factors of FIR and more comprehensive indicators to be studied. Another limitation of this study is that more segments and paravertebral muscle‐related aspects such as lumbar square muscles were not included for study. In addition, the CLBP patients included in this study all came from the same hospital, and there is a lack of extensive, nationwide, large‐sample, multigroup data.

This study measured the L2–S1 segment, excluding the L1‐L2 segment. Although this decision was based on the aforementioned considerations of imaging feasibility, it may have limited our comprehensive understanding of the pattern of changes in the paraspinal muscles of the upper lumbar spine. Future research could explore optimizing the scanning sequence or utilizing other imaging planes to include additional segments.

This study employed a retrospective cross‐sectional design, with sample size determined based on available cases within the established study period. No prior power analysis was conducted to calculate the minimum sample size required to detect specific effect sizes. This may impact the statistical interpretability of findings, particularly those exhibiting weak or no correlations. Future prospective studies should incorporate rigorous sample size estimation during the design phase. Additionally, intrarater and inter‐rater reliability for Pfirrmann grading was not calculated using intraclass correlation coefficients (ICCs), despite consensus‐based grading by two experienced spine surgeons. This represents a methodological limitation of the present study, and ICC analysis will be included in future research.

## 5. Conclusions

We analyzed the groups more prone to low back pain according to age, sex, and fatty infiltration site and excluded factors not related to FIR such as BMI and location of low back pain; in addition, we found an indirect link between IDD, the degree and effect of low back pain, and FIR, which provides a new way of thinking about the assessment of patients with low back pain and the evaluation of the effectiveness of treatment and a new angle of exploration of IDD.

**Nomenclature**
BMI: Body Mass Index
CLBP: Chronic Low back pain
CSA: Cross‐sectional area
ES: Erector spinae
FIR: Fatty infiltration rate
FSU: Functional spinal unit
MF: Multifidus
MRI: Magnetic resonance imaging
ODI: Oswestry Disability Index
PM: Psoas major
VAS: Visual Analog Scale

## Author Contributions

Conceptualization: Bo Gao and Xiwen Liu; data curation: Xin Lei and Fang He; investigation: Weiyi Wang and Xin Lei; visualization: Weiyi Wang and Jintao Dong; validation: Simin Chi; writing‐original draft: Weiyi Wang; writing‐review and editing: Weiyi Wang and Guanyi Wang; supervision: Bo Gao, Xiwen Liu, and Zhuojing Luo.

## Funding

This study was supported by the General Program of the National Natural Science Foundation of China (Grant No. 82572806); the Key Research and Development Projects of Shaanxi Province (Grant No. 2024SF‐ZDCYL‐04‐03); and the Rapid Response Special Project of the Medical Staff Promotion Program of Xijing Hospital (Grant No. XJZT25KX02).

## Ethics Statement

The study protocol was approved by the Ethics Committee of Xijing Hospital, Air Force Medical University (Approval No. KY20242136).

## Consent

Informed consent was obtained from all subjects involved in the study.

## Conflicts of Interest

The authors declare no conflicts of interest.

## Data Availability

Data used to support the findings of this study are available from the corresponding authors upon reasonable request.
